# Automatic recognition of therapy progress among children with autism

**DOI:** 10.1038/s41598-017-14209-y

**Published:** 2017-10-24

**Authors:** Agata Kołakowska, Agnieszka Landowska, Anna Anzulewicz, Krzysztof Sobota

**Affiliations:** 1 Gdańsk University of Technology, Faculty of Electronics, Telecommunications and Informatics, Gdańsk, Poland; 2 Harimata Sp. z o.o., Kraków, Poland; 3 Jan Matejko Academy of Fine Arts, Kraków, Poland

## Abstract

The article presents a research study on recognizing therapy progress among children with autism spectrum disorder. The progress is recognized on the basis of behavioural data gathered via five specially designed tablet games. Over 180 distinct parameters are calculated on the basis of raw data delivered via the game flow and tablet sensors - i.e. touch screen, accelerometer and gyroscope. The results obtained confirm the possibility of recognizing progress in particular areas of development. The recognition accuracy exceeds 80%. Moreover, the study identifies a subset of parameters which appear to be better predictors of therapy progress than others. The proposed method - consisting of data recording, parameter calculation formulas and prediction models - might be implemented in a tool to support both therapists and parents of autistic children. Such a tool might be used to monitor the course of the therapy, modify it and report its results.

## Introduction

Autism is a complex developmental disorder that influences the ability to communicate and learn. Autism is nowadays a growing challenge, as the number of children diagnosed with autism is rising worldwide^1^. The disorder exhibits a spectrum of symptoms that might range from mild to severe in a particular case, varying from skill to skill and from child to child. This makes diagnosis and therapy progress evaluation difficult and at the same time crucial for the effectiveness of the therapy. Early identification and proper therapy translates into a greater chance for preventing a person with autism from social exclusion. Therefore, any idea that might improve therapeutic practice is worth investigating. This paper presents one such idea, which incorporates the analysis of behavioural characteristics observed in autistic children while they play specially designed tablet games. This interdisciplinary study combines computer science and special pedagogy, by applying computer technologies and machine learning methods in the process of therapy for autistic children.

Computer technologies may support both the diagnosis of autism and related therapy, although most solutions so far refer to the process of therapy. There are numerous applications designed for individuals with autism. These applications focus on particular issues by teaching specific skills–e.g. expressing needs, learning certain behaviours^2^, improving verbal communication, answering questions, interacting with other people in typical situations^3^, recognizing and expressing emotions^4–6^. These solutions take advantage of the fact, that children with autism are usually enthusiastic about tasks supported by computer technology, which offers a predictable framework without causing stress^2^.

Another group of tools is designed for therapists. One of the most commonly used solutions are computer versions of standardized questionnaires that evaluate an individual’s state^7^. Another popular way of supporting the therapy is to provide the experts with video recordings of the children’s behaviour. However, there are also some more sophisticated solutions, which may be used not only in therapy but also in the diagnostic process. Some of them are based on eye tracking and they reveal that people with autism rarely focus their attention on the area around the eyes when compared to other parts of the face. This idea can be implemented in the early diagnosis of ASD^8,9^. Others apply camera-based motion capture and automatic analysis of recordings presenting individuals performing tasks that are commonly evaluated by specialists^10^. Another interesting idea is based on the analysis of individual hand movements while indicating objects on a touch screen^11^. Moreover, machine learning algorithms have been applied to predict the state of a person on the basis of information taken from commonly used diagnostic questionnaires^12,13^. These are usually experimental methods used in laboratories, often tested on small groups. In many cases, the use of these technologies requires specialized equipment, which constitutes their major drawback–their limited availability. These and similar solutions are in the process of continual improvement and validation. Therapeutic tools based on computer technologies have a chance to become prevalent in institutions, if they are implemented on commonly used devices such as, for example, tablets. Specially designed applications running on these devices may not only monitor the accuracy of tasks solved by the user, but also the way they are solved thanks to the fact that tablets are equipped with a number of sensors–i.e. touch screen, accelerometer, gyroscope - able to record behavioural characteristics continuously.

The behavioural characteristic is a parametric description of the actions, reactions or functioning of a human, under normal circumstances or as a response to a specific stimulus. It may be easily affected by numerous factors–e.g. the emotional states of an individual, intentional modification or changing environment. On the other hand, the main advantage of behavioural characteristics is their natural, usually unobtrusive method of data collection^14^. There are numerous applications incorporating behavioural parameters to authenticate users depending on the way they use input devices^15^, speak^16^ or walk^17^ etc. They may also be applied to the recognition of emotional states^18^ and even gender^19^. In the case of autism behavioural phenomena are noted by human observers. This is a widely accepted method for analysing therapy progress^20^ and as criteria in autism diagnosis–e.g. according to DSM-V^21^. The automated analysis of behavioural data in a tool supporting diagnosis or therapy of children with ASD is not a common idea. There are even fewer examples of using mobile devices for this purpose. There are several behavioural characteristics that might be observable from the perspective of a tablet device. Controlling tablet applications requires specific motor skills. Therefore, these mobile devices would seem to be appropriate for this particular task.

Although autism is primarily associated with social and emotional impairment, it also affects other skills such as fine and gross motor skills, speaking, self-reliance and the ability to follow instructions. Children with ASD present significant motor deficiencies, which are one of the first symptoms that may be observed in early infancy, even before a medical diagnosis is possible^22^. These impairments are manifested in whole body control and the sequencing of movements of the trunk, head and limbs to control balance and postural changes^23^. Numerous studies have been performed to analyse the differences in kinematic profile between individuals with autism and those who develop typically^24–27^. This is manifested, for example, in the way a particular movement is prepared^27^, in the velocity, acceleration and jerks while specific types of arm movements are performed^24^, or in different distributions of grip force over time^25^. One of the latest research studies presents promising results on the automatic diagnosis of ASD on the basis of data gathered from tablet sensors^26^. The authors of this solution managed to identify children with autism with up to 93% accuracy. The way children perform gestures such as tap, swipe or finger move turned out to be significant in discriminating between individuals with autism and their typically developing peers.

There are studies confirming the fact that cognition and action are mutually dependent and they form a system around which adaptive behaviours develop^28^. Therefore, some researchers investigate the impact of motor problems observed among individuals with autism on other areas of their development^24,25,29,30^. Motor skills may affect the development of language and in this way they drive social development and interactions^31,32^. One of the studies revealed that fine motor skills are predictors of adaptive behaviour composite, daily living skills, adaptive social and communicative skills, whereas gross motor skills turned out to be indicators of daily living skills^29^. Social deficits, on the other hand, may affect another domain–e.g. some relationship has been found between impairment in social interaction skills and challenging behaviours for children with ASD^30^. Another study suggested that the atypical kinematic profile, which explains perceptual and motor impairments, might have some influence on higher level social problems–e.g. the inability to communicate through gestures or facial expression^24^. The authors of this study have even identified a positive correlation between the anomalies in movement ascertained and autism severity measured by the Autism Diagnostic Observation Schedule (ADOS)^33^. They also posed a challenging research question as to whether an improvement in kinematics could improve social perception and interactional abilities.

The mentioned results and open questions motivated the authors of this study to verify a notion that the changes in motor skill measurements taken while playing specially designed tablet games together with changes of game flow data may provide valuable information on the progress of therapy. More specifically, these measurements might be considered as therapy progress indicators. Moreover, due to the relationship between motor skills and other domains of development, the study includes the analysis of the possibility of monitoring progress not only in motor skills, but in other developmental areas as well.

The research presented in this paper has been conducted within the AUTMON project entitled “Automated progress monitoring for children with autism spectrum disorder”^34^. The aim of this study was to answer the question as to whether or not it is possible to recognize therapy progress in selected areas of a child’s development on the basis of behavioural characteristics observed from tablet sensors during gameplay. Moreover, if the answer for the above question is positive, the research should identify a set of parameters which might be used as indicators of the progress in mastering particular skills. Although controlling a tablet application via its sensors seems to be mainly associated with motor skills, there are grounds for considering progress made in other areas of development as well.

In the study presented, children with autism were measured during several subsequent sessions carried once a month during a half year period. The data were gathered via specially designed tablet games. A number of parameters were calculated on the basis of raw data. The data samples were assigned labels according to the evaluations of therapists in the following ten areas of development: communication skills, fine motor skills, gross motor skills, following instructions, self-reliance, social and emotional skills, stereotypical behaviours, reaction to stimulation, attention control, challenging behaviours. A number of machine learning methods have been used to verify the idea of therapy progress recognition. The methods included supervised training algorithms applied to train two-class classifiers differentiating between two states: *progress* and *no progress*. The experiment design and methodology has been described in detail in the Methods section, while detailed study results are included in the Results section.

## Methods

The aim of the research project was to verify the idea that it is possible to recognize progress in the therapy of autistic children on the basis of behavioural data observed from specially designed tablet games. Another goal was to search for parameters that are easy to calculate and provide the maximum information gain in terms of the evaluation of certain skills. To achieve these goals, a longitudinal study was planned. The research methods used in this study include: interviewing therapists in order to prepare the evaluation questionnaire used to label data samples, gathering data on child-tablet interaction and labelling it with information on the child’s progress, data analysis using a machine learning approach and finally a post-hoc questionnaire completed by the therapists to evaluate the usefulness of the study results. The experimental protocols employed were specially designed and conducted in accordance with the Declaration of Helsinki and approved by the Jagiellonian University Ethical Committee. The methods were carried out in accordance with the relevant guidelines and regulations.

### Participants

40 children aged 37–83 months (mean 60 months, standard deviation 14 months), clinically diagnosed with Childhood Autism (ICD-10, World Health Organisation) were included in the study. Of these, 9 were female and 31 male. The children were recruited from 10 special therapeutic centres in Poland and selected by clinicians. Diagnosis was obtained by medical practitioners prior to the study. Exclusion criteria included co-morbid impairments (uncorrected vision and hearing, disabilities impairing motor skills). Not all children completed the study and contributed to the final data. The drop-out resulted from absence in consecutive sessions. The absence rate resulted from temporary illnesses and was typical for the participants age range. The number of monthly sessions per child varied from 1 to 7. At least two sessions were required to include a child’s data in the final datasets. Prior to the study, the children’s parents gave written informed consent for their child’s participation.

### Data collection tool

**Figure 1:**
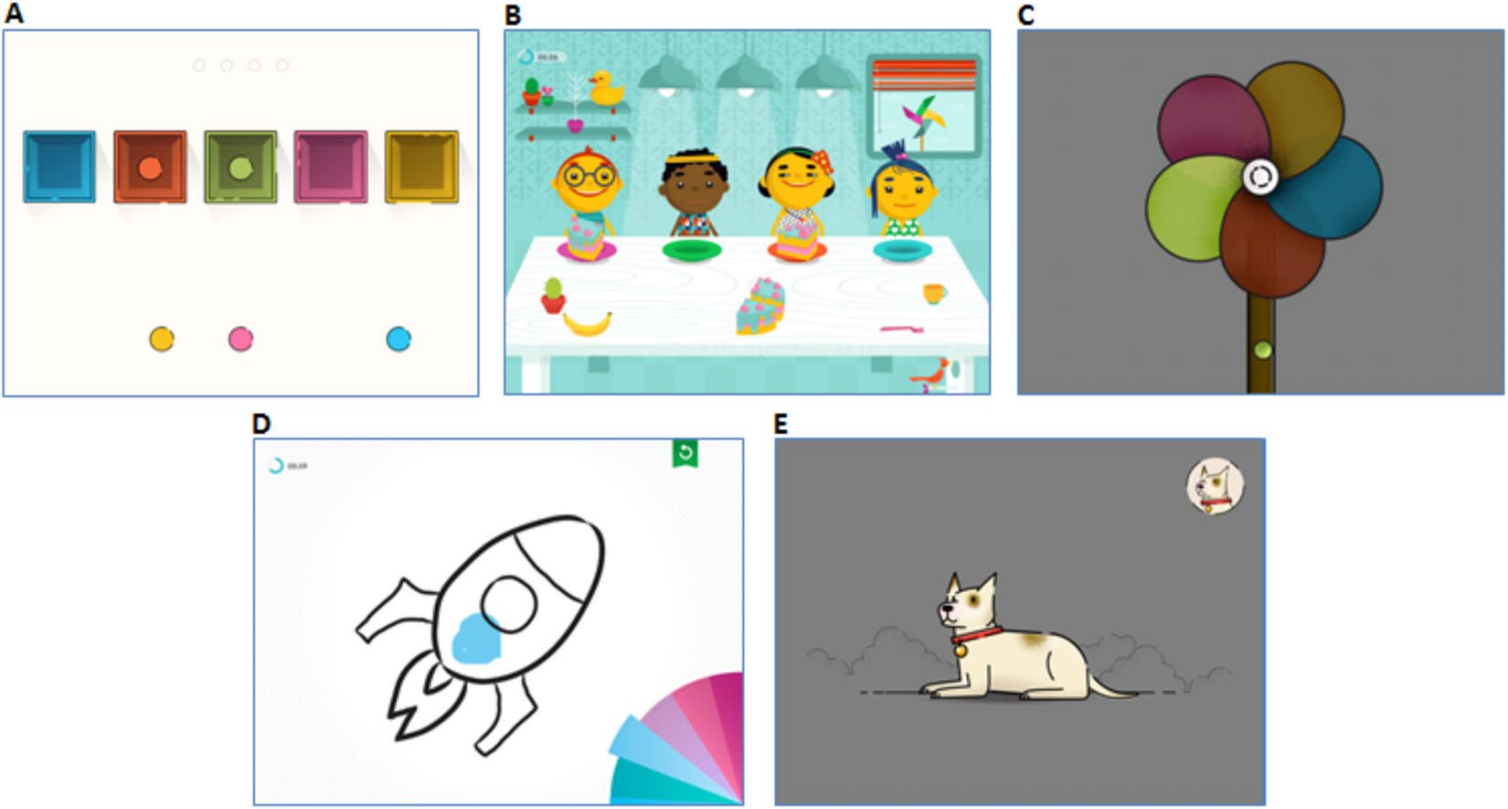
Screenshots from the games used as a data collection tool: (**A**) Boxes, (**B**) Sharing, (**C**) Pinwheel, (**D**) Creativity, (**E**) Cat and Dog.

To collate the experimental data, a tool consisting of five games presented in Fig. 1 was designed and implemented. The application was implemented for iPad devices and all the data in the experiment were collected using the iPad mini. Two of the games (*Sharing*, *Creativity*) were designed by Duckie Deck Development (www.duckiedeck.com) and commercially available as educational games for children aged 2–5 years. Three others (*Boxes*, *Pinwheel*, *Cat and Dog*) were designed within the AUTMON project.

*Boxes* is a simple educational drag-and-drop game. The goal is to place the balls into boxes by matching corresponding colours (Fig. 1A). A child should touch and then drag all five balls into their proper box. It is very simple, yet allows to measure colour recognition, simple instruction understanding and matching ability. It also becomes clear if a child can use the touch screen (swipe movements) properly. The game time is not limited in this case, although four rounds were planned. Each round requires 5 balls to be properly placed. This game was primarily designed as a warm-up, but it turned out that its level of difficulty was appropriate for children with a developmental age of 3-4 years^35^.

*Sharing* is a game where food should be divided among four animated children (Fig. 1B). The player has to tap on the displayed food article (watermelon, apple, cake) and drag pieces over to four plates. The game consists of a learning phase and an identical test phase. This task requires understanding more complex instructions, motor precision, perseverance (the game is all about sharing for 5 min) and to some extent attentional control, as the screen is filled with clickable distractors. The distractors are aimed to draw the players’ attention and prevent them from completing the tasks. The task cannot be completed if the food pieces are allocated unevenly (if some children are left without their portion) or if the non-verbal cues given by characters are not read (if a child doesn’t get his/her portion, his/her face turns sad, in contrast to the happy faces of those who get a piece)^35^.

*Pinwheel* is a game that requires some flexibility in terms of the unusual manipulation of the tablet, different than those learned in previous games. Performance in *Pinwheel* is determined by the efficiency and speed of learning new ways to use objects, fine motor proficiency, instruction understanding, executive control and attention, inhibition, colour recognition and matching as well as many other basic cognitive abilities. A pinwheel slowly spins while a colour ball balances at the base of its stem (Fig. 1C). The child has to flip the tablet (previously only touch screen data were gathered, whereas now the accelerometer and gyroscope provide the data) precisely to move the colour ball to the corresponding pinwheel petal. Should the ball fall off the stem, the child has to start over with the next attempt, and the same happens if the ball lands in the wrong petal (for example, blue ball hits the yellow petal). Patiently waiting, anticipatory movement planning and ball control can be challenging for younger users, so the game is recommended for older children^35^.

*Creativity* game requires drawing and colouring pictures (Fig. 1D). The child has to exhibit a good understanding of the instruction, stability and precision of movement, as well as an ability to imitate (mimic) the lines displayed as a prompt. Each step of drawing a picture is briefly displayed on the screen and child’s task is to run a finger along the dotted line. In the end, the child gets to colour the finished picture–leaving even more movement patterns for analysis. This game turned out to be the most rewarding for most children^35^.

The *Cat and Dog* game was intended to be used by older children. Typically developing 3-4 years old children are not ready to proceed with this cognitively demanding task. A series of stimuli is presented randomly at a fast pace and the child has to react to target stimuli while refraining from reacting to distracting stimuli. For example, when a dog is presented in the corner of the screen, as shown in Fig. 1E, the child has to tap the screen as fast as possible when a dog appears in the centre or if a barking sound is heard and not react to a cat or meowing. This task demands prolonged alertness, fast decision making, good perceptual and attentional skills and executive control of intentional actions. It can be used to measure impulsivity and control processes. As this game can be frustrating for some children, it is advised to omit the game if the child exhibits any loss of interest or negative emotions (usually auto-stimulatory behaviours)^35^.

Table 1 presents different skills required by each game. A thorough study was conducted to evaluate several usability factors–i.e. understanding, engagement and enjoyment, shown by children while playing particular games^35^. This study revealed that three of the games: *Sharing*, *Creativity* and *Boxes* were mostly understandable for the children. *Sharing* seemed to be their favourite game. *Cat and Dog* and *Pinwheel* turned out to be less intuitive and half of the children refused to use them. However, once a child understood the game, it immediately became engaging. Another interesting observation was that although some of the children remembered the games after a month (the average time lapse between the recording sessions), the data did not appear to have been influenced by the history effect. This observation would appear to be significant if the games are to be used as a tool for monitoring therapy progress.

**Table 1:** Skills required by the games.

Required skill	Boxes	Sharing	Pinwheel	Creativity	Cat and Dog
simple instruction understanding	x			x	
complex instruction understanding		x	x		x
perseverance		x	x		x
patiently waiting			x		
attention control		x	x		x
executive control			x		x
fast decision making			x		x
imitating				x	
reading mimics		x			
color recognition and matching	x		x		
anticipatory movement planning			x		
movement precision	x	x	x	x	
movement stability				x	

### Data collection protocol

Each child played the games several times during a 6-month period. Subsequent sessions were carried once a month. The data from each session were recorded, along with a questionnaire completed by therapists and followed by comments from the researchers. The children’s reactions to the tablet games and the study were very diverse. Some of them had difficulties in accepting the presence of a researcher and were unable to start playing, while others started to accept the situation during the second or the third session. Some children were so keen on technology, tablets and games, that the novelty of the situation was not disturbing at all and they immediately got involved in the gameplay and did not want to finish it after the time limit. The researchers took notes on each session, including information on the child’s eagerness, engagement, understanding along with any problems.

Each experimental session consisted of a training phase and the actual game. During the training phase, a child might become acquainted with the game concept. During the whole training, the therapists had to instruct the children and used either verbal or non-verbal guidelines. At any time, the training session could be terminated by the experimenter or skipped, if not required.

After finishing the training phase, the actual game, which was used to collect the measurements, began. In order to obtain undisturbed results, during this phase, the therapist could not interact with the child. Similarly, during the training phase the session could be terminated if or when the child lost interest in interacting with the game. 40 children with autism took part in the study, but only about 30 of them delivered amounts of data substantial enough to be included in the experiment.

### Data labelling

Each sample of data collected had to be labelled so that the supervised machine learning algorithm could be applied, which could then build models used to predict the progress of therapy. These labels were assigned according to the therapists’ evaluations.

During the study we cooperated with 10 therapy centres located throughout Poland. The centres differed a great deal in terms of the timing, scope and precision of the children’s assessment - some evaluated a child’s progress every half a year and only a descriptive language evaluation was conducted, while others evaluated individuals every week, using precise, although narrow, scales. As we needed to find a compromise between these extremes, a special questionnaire was designed for this study and used in order to ascertain the children’s progress. The questionnaire was completed during each measurement session by a therapist who worked with the child and knew him/her very well. The progress was estimated using a 74-item scale centred around the following ten skill areas:

- communication skills (spontaneous eye contact, speech understanding, reacting to name, indicating, imitating adults or other children, adequate gestures, alternative communication skills, ability to use speech, greeting and saying good bye, asking and answering questions, spontaneously starting to communicate with adults or other children, expressing physiological needs, expressing other needs and preferences, maintaining a conversation);
- fine motor skills (holding pencil, colouring, tracing a drawing, cutting, grabbing small objects, building a tower of blocks, putting beads onto a necklace, fastening buttons);
- gross motor skills (standing on one leg, jumping on one or both legs, squats, climbing stairs, throwing a ball at a target, kicking a ball, running, riding a bike, climbing a chair/sofa);
- following instructions (both simple and more complex instructions);
- self-reliance (dressing, eating, cleaning up, using a toilet, washing hands, tooth brushing);
- social and emotional skills (reciprocal smile, contacting adults or other children, sharing with adults or other children, recognizing others’ emotions, understanding their own emotions, joining other children spontaneously while sitting in a circle or around a table, asking for help, empathic reactions to others’ emotions, taking part in a symbolic play);
- stereotypical behaviours (motor stereotypes, echolalia, routine behaviours, reaction to an announced or unexpected change in a given activity routine or in everyday routine activities, reaction to novelty),
- reaction to stimulation (overactivity or understimulation in the area of motion, eyesight, hearing, taste, smell and touch);
- attention control (following others’ indication, indicating, focusing on a given task, focusing on a therapist while he/she is talking, ability to move from one activity to another);
- challenging behaviours (aggressive or autoaggressive behaviours, crying, screaming, apathy, laugh, escaping, emotional lability, not accepting refusals, stimulating behaviours, problems with calming down).

The therapists evaluated children in these ten different areas of development. Each area included several elements to be taken into account and each of them was evaluated by a therapist using a few point scale. Each element rating was then transformed into a number from [0, 100] range. The average derived from the values of several elements gave the evaluation for a given area. If this value increased from one session to another then we assumed there was some progress in the given area of development; otherwise we assumed no progress. The *progress*/ *no progress* information constituted classification labels for our training data. The data from the questionnaire labels were assumed to be the ground truth in the study, although such approach is not free from limitations.

### Feature extraction

The raw data recorded during gameplay were processed to extract a number of parameters defining each game played by each child. These parameter values constituted training vectors used to build models for therapy progress recognition.

The idea of the parameters extracted from the games arose from studying and identifying kinds of movements responsible for differentiating between children with autism and children developing typically while they play tablet games^26^. That research revealed that individuals with autism use a greater force of impact and lateral movements, which could be measured by the accelerometer. Moreover, the different distribution of forces applied on the device were revealed through the gyroscope data. Several other characteristics were identified after analysing the data from touch screen sensors–i.e. greater gesture velocity, larger areas occupied by gestures and faster screen taps. Some of the features showed large variations among the children with autism.

These observations motivated us to investigate how these parameter values change during a specified period of a child’s therapy. Our study presented in this paper was conducted by using data gathered from tablet sensors, but it focuses on analysing their changes across time and the ability to measure a child’s progress. Each of the five games presented was a source of valuable information both on the game flow and the way the tablet was operated.

Three types of features may be distinguished depending on their source of information. The first type are features describing game flow. They present how well a child copes with a given task, e.g. correctness of actions, time needed to solve a task. The second type are features calculated on the basis of data coming from touch screen. They present motor characteristics, e.g. velocities of movements, shapes occupied by gestures. The third type are features from inertial sensors, i.e. accelerometer and gyroscope. These features describe other motor characteristics, e.g. force put in different directions, jerks.

The *Boxes* game yielded data about the game flow such as the colours of balls placed in particular coloured boxes and the way touch screen is used–i.e. the way a ball is moved across the screen35. The first group of features relates to the game flow and contains the following attributes:

- total game time,
- the total number of ball moves (not only those reaching a box),
- for each of the five balls, the number of successful placements in a box (usually this is 4; only if a child does not complete all four rounds could it be lower than 4),
- for each of five balls the number of unsuccessful placements in box–i.e. a box of the wrong colour,
- the number of moves divided by the total game time calculated for successful, unsuccessful and the total number of moves.

The second group consists of the parameters calculated on the basis of raw data from the touch screen. All the ball moves ending in a box, both successful and unsuccessful, are taken into account. Moreover these statistics are calculated not only for all moves, but also separately for a subset of short ones. The group contains the following features:

- the number of paths,
- the length of the shortest possible path to the length of the actual path (average, minimum, maximum),
- moving speed (average, minimum, maximum).

The *Sharing* game revealed the following characteristics of the game flow, which mainly describe whether a child follows the game’s rules:

- total game time,
- the number of all actions divided by the total time,
- the number of successful actions divided by the total time,
- average length of one round
- the number of food piece movements (from table or plate to plate) divided by the total time,
- the number of other object movements (from table or plate to plate) divided by the total time,
- the number of events a distractor was used divided by the total time (calculated for different groups of distractors: lamps, shelf objects, bird, pinwheel and all together),

Several more parameters, calculated for moving food pieces from table to plates, describe the way a child executes drag gestures. These statistics were extracted separately for each animated character and for all of them. This set contains the following attributes:

- the ratio of the shortest possible path length to the actual path length (average, min, max, median),
- moving speed (average, min, max, median).
- The *Pinwheel* game’s set of features include the following attributes that relate to the game flow^36^:
- total game time,
- the number of successful or unsuccessful petal hits divided by the total time,
- the number of times a ball falls off the screen divided by the total time,
- the number of all rounds (balls) divided by the total time.

The second group consists of the following parameters:

- the number of screen touches divided by the total time,
- mean reaction time for successful, unsuccessful and all hits.

The features in the third group describe the tablet orientation during the game. They are calculated on the basis of data from the gyroscope–i.e. Euler angels known as roll and pitch which change while tilting a tablet forward and backward or left and right. Extracting these parameters requires the definition of a threshold parameter. A tablet is considered to be tilted when a roll or pitch value exceeds the threshold. The following set of tilt parameters is extracted for all tilts - i.e. forward (to move the ball towards the flower), backwards (to move the ball away from the flower), left and right:

- the percentage of time the tablet was tilted
- the number of tilts divided by the total time,
- maximum tilt (mean, standard deviation, median),
- tilt oscillations measured as the number of local extremes for a tilt (mean, standard deviation, median).

The *Creativity* game delivered the largest amounts of raw data. The first subset of extracted parameters describing the game flow is as follows:

- total game time,
- the number of images selected,
- the number of completely outlined imaged,
- the number of images, for which colour filling began.

The second group of characteristics describing the way a child draws contains the following parameters:

- the speed of path drawing (mean, standard deviation, min, max) calculated separately for contour paths, colour filling paths and all paths together,
- the length of colour filling paths (mean, standard deviation, min, max, median),
- horizontal and vertical range of colour filling paths (mean, standard deviation, min, max, median),
- the number of colour changes per image,
- the intensity of drawing measured by changes of X (for horizontal paths) and Y (for vertical paths) acceleration sign in time,
- the pressure intensity measured by changes of Z acceleration sign in time,
- the percentage of time the acceleration exceeded a threshold value (separately for X, Y, Z directions).

*Cat and Dog* game, which requires only tap gestures at some moments, provided us with the following set of features:

- total game time,
- the number of touch answers divided by the total time calculated for correct and incorrect answers,
- the number of screen touches over an unexpected region divided by the total time,
- the number of missed touches divided by the total time,
- the percentage of touch answers,
- the percentage of restrain events (both correct and incorrect answers without touching the screen),
- reaction time (mean, standard deviation, min, max) calculated for correct touch answers (separately for image and for sound) incorrect touch answers (separately for image and for sound) and all touch answers.

The source of the features was either the game flow, touch screen, accelerometer or gyroscope. All games delivered parameters from the game flow and the touch screen, *Pinwheeel* additionally from the gyroscope and *Creativity* from the accelerometer. All feature values were normalized to [0, 1] range. The total number of extracted features was 181 and this varied from 27 to 44, depending on the game. To reduce this number, which seems to be high if compared to the number of training samples, principal component analysis (PCA) was performed. The experiments were performed for different types of training data sets–i.e. raw data, raw data + PCA, normalized data, normalized data + PCA. Different transformations resulted in different numbers of features. Table 2 presents the numbers of raw features extracted from each game and the numbers after the transformations.

**Table 2:** Numbers of features calculated on the basis of data from the games.

Game	Number of original features	Reduced number of features after performing PCA	
		on original features	on normalized features
Boxes	29	4	12
Sharing	44	9	20
Pinwheel	41	5	19
Creativity	41	9	20
Cat and Dog	27	4	16

### Classifier training

The stated problem was to train a classifier to recognize whether or not a child has demonstrated progress in different areas of development. Thus, the parameters described previously were not directly taken to constitute feature vectors, but the differences between their values from different sessions were taken into account. Two types of recognition tasks were analysed. The first one was based on the analysis of the differences between the first and the last session for each child. The second task was based on the analysis of the differences between two subsequent sessions. In both cases, the feature vectors consisted of differences in the values calculated for all the previously described parameters collected either for two subsequent sessions or for the first and the last one. The main difference in these two tasks was the size of the training set. While analysing the differences between the first and the last session, the maximum possible number of feature vectors was equal to the number of children taking part in the study. In the second case it was possible to extract a few vectors for each child, depending on the number of sessions a child took part in.

According to the *no free lunch* theorem, no machine learning algorithm is universally better than any other^37^. The choice of method depends on the type and amount of available training data and usually a number of methods are applied to solve a particular problem. The methods that are being explored extensively nowadays are deep neural networks. Deep learning requires large amounts of training examples, which were not collected in our case. Therefore, it was not possible to apply these types of algorithms. The authors relied on traditional algorithms used in the case of supervised training tasks–i.e. tasks based on data consisting of samples accompanied by desired outputs. These outputs, in our case, were the progress labels assigned on the basis of questionnaires completed by the therapists. A number of machine learning methods used for supervised training tasks were applied to build a classifier. Representatives from different groups were tested: single classifiers and combinations of them. The authors anticipated using methods that would combine more classifiers, such as for example bagging^38^, random forests^39^, rotation forests^40^ or AdaBoost^41^, that might cope with the problem of a reduced quantity of training data through their use of many modified versions of training data sets to build multiple models. The final decision is then made on the basis of a voting procedure. The training set modifications are obtained either by random sampling of the examples and features or by adding weights to the samples. Random forests and rotation forests are combinations of decision trees. Bagging and AdaBoost may incorporate different classifiers. In this study they were also applied as sets of decision trees. Single decision trees, known from their simplicity and legibility, which is crucial in some applications, but sometimes also problematic in terms of achieving a low generalization error, were also implemented^42^. This enabled us to compare the results obtained using single models and their combinations. Several other single models were applied in the experiment, among them feedforward neural networks famous for their ability to solve complex nonlinear problems^43^ and methods based on evaluations of probability distributions - e.g. Bayesian networks an naive Bayes^44^. The latter, in spite of its simplifying assumption on feature independence, often leads to satisfying results in many known applications.

For each game, ten independent classification problems were solved, one for each area of development. To perform the experiment, WEKA software was used^45^. To test the classifiers, a 10-fold cross-validation method was applied, which enables error estimation even when the number of training data is not large enough to have a separate testing data set. The training data were divided into 10 subsets in a stratified manner–i.e. preserving equal or almost equal class distributions in all subsets. Nine of the subsets were designed for training, one for testing, changing the roles in subsequent iterations. The whole 10-fold cross-validation procedure was repeated 10 times, which allowed us to calculate the standard deviation and the confidence intervals of the error rates. It is a standard approach applied in order to reduce the effect of random variation in choosing the folds and thus obtain a more reliable error estimate^46^. The confidence interval, in the case of repeated cross validation, may be calculated applying the t-test statistic with the number of degrees of freedom equal to the number of repetitions minus one^47^. Moreover, precision (the probability that a particular prediction is correct), recall (the probability of correct prediction in a particular class) and F-measure (harmonic mean of precision and recall) for both the *progress* and *no progress* classes were estimated^48^.

## Results

### Therapy progress recognition

Table 3 presents the best results obtained for each area. It includes the overall accuracy together with its 95% confidence interval and other parameters, such as precision, recall and F-measure estimated for both classes. If, for a given area of development, the accuracy had not exceeded 60% the result for this area would not have been presented in the table. The presented results refer to the recognition of progress between the first and the last session a child took part in - i.e. after a few month period.

**Table 3:** The best results obtained for each game and each area of development.

Area of development	Acc. [%]	95% Conf. int. [%]	Precision [%]		Recall [%]		F measure [%]		Method
			P	N	P	N	P	N	
**Boxes**									
communication skills	78.97	±3.38	81.05	75.00	86.12	67.53	83.51	71.07	Bayesian network
	78.28	±3.08	79.47	76.00	86.34	66.26	82.77	70.80	Decision tree
fine motor skills	81.38	±3.93	83.16	78.00	87.81	71.57	85.42	74.65	Bayesian network
gross motor skills	60.36	±2.15	57.50	62.50	54.08	65.91	55.74	64.16	Decision tree
self-reliance	73.93	±3.70	82.22	59.00	78.37	65.26	80.25	61.97	AdaBoost
	73.21	±2.70	87.22	48.00	75.20	67.50	80.77	56.10	Random Forest
social and emotional skills	65.86	±5.46	72.78	54.55	73.02	54.23	72.90	54.38	AdaBoost
stereotypical behaviours	73.45	±0.00	84.29	45.00	80.00	54.19	82.09	49.17	Decision tree
attention control	68.28	±3.89	74.71	59.17	72.51	62.64	73.59	60.85	Bagging with trees
challenging behaviours	66.90	±5.97	73.85	61.25	61.02	74.55	66.82	67.25	Random Forest
	66.21	±3.49	66.92	65.63	61.95	70.96	64.34	68.19	Decision tree
**Pinwheel**									
communication skills	77.60	±1.48	80.95	72.61	82.00	71.00	81.44	71.72	Decision tree
fine motor skills	72.40	±4.94	78.88	60.69	81.18	53.75	79.62	55.86	Neural network
gross motor skills	78.80	±4.68	71.96	81.87	58.75	88.24	64.37	84.88	Decision tree
following instructions	70.00	±4.31	73.20	67.20	67.69	72.50	70.25	69.68	Naive Bayes
stereotypical behaviours	80.80	±1.81	83.16	74.52	90.00	61.25	86.43	67.14	Neural network
reaction to stimulation	72.08	±3.73	66.59	79.85	80.91	64.62	72.82	71.11	Naive Bayes
**Sharing**									
communication skills	77.86	±2.35	81.70	68.29	86.84	58.89	84.16	63.10	Decision tree
fine motor skills	81.79	±1.45	87.23	71.11	85.79	73.33	86.47	72.07	Neural network
gross motor skills	79.26	±2.56	77.50	80.31	69.09	86.25	72.98	83.15	Neural network
reaction to stimulation	79.26	±3.11	68.93	83.14	56.25	88.95	60.92	85.78	AdaBoost
attention control	81.79	±3.28	91.62	73.35	75.00	90.83	82.40	81.10	Neural network
challenging behaviours	65.71	±3.65	63.70	67.52	60.77	70.00	62.04	68.62	Neural network
**Creativity**									
communication skills	67.19	±1.18	68.33	65.15	77.78	53.57	72.74	58.77	Naive Bayes
fine motor skills	76.88	±3.19	79.63	72.69	82.11	69.23	80.82	70.84	Desision tree
following instructions	64.69	±1.51	72.53	59.58	54.12	76.67	61.95	67.04	Naive Bayes
social and emotional skills	70.32	±3.73	71.07	69.86	77.06	62.14	73.78	65.51	Decision tree
reaction to stimulation	80.00	±2.12	71.53	89.04	86.92	75.00	78.40	81.34	Bayesian network
attention control	67.50	±2.62	71.20	64.06	65.29	70.00	68.07	66.86	Decision tree
challenging behaviours	66.56	±4.08	62.26	70.00	60.71	71.11	61.32	70.46	Rotation forest
**Cat and Dog**									
communication skills	85.00	±2.68	84.93	86.67	94.17	66.67	89.26	75.03	Decision tree
fine motor skills	66.11	±5.11	63.24	70.91	75.56	56.67	68.76	62.82	Bayesian network,
gross motor skills	69.44	±3.86	60.30	75.60	61.43	74.55	60.65	74.95	Bayesian network
self-reliance	72.22	±3.74	68.86	75.56	70.00	74.00	69.18	74.58	Neural network
social and emotional skills	88.89	±0.00	84.62	100.00	100.00	71.43	91.67	83.33	Decision tree
stereotypical behaviours	74.12	±2.94	78.57	68.57	77.00	70.00	77.66	69.08	Neural network
reaction to stimulation	78.24	±3.46	75.82	82.14	86.67	68.75	80.84	74.76	Decision tree
attention control	67.22	±9.25	69.05	64.33	77.00	55.00	72.56	58.81	Rotation forest

Precision, recall and F-measure are given for *progress* (P) and *no-progress* (N) class.

Communication skills turned out to be the area in which all games, except for *Creativity*, successfully coped with the task of progress recognition. The best results of around 85% were obtained by the *Cat and Dog* game. This observation may arise from the fact that *Cat and Dog* turned out to be the most difficult for the children. Some of them refused to play the game. However, once they understood the rules, they enjoyed playing it. Slightly lower, but all on the same level, results of between 77% and 79% were obtained for *Boxes*, *Sharing* and *Pinwheel*.

In the case of fine motor skills, the *Boxes* and *Sharing* games appeared to be the most appropriate and achieved more than 81% accuracy. These two games require movement precision while dragging objects to targets, so this was expected. Unexpectedly, the *Creativity* game, which requires drawing and colouring images, did not yield such good results in the area of fine motor skills.

Two of the games, *Sharing* and *Pinwheel*, appeared to be good at recognizing progress in gross motor skills. The achieved accuracies were about 79% in both cases. *Pinwheel* was the only game requiring some gross motor abilities while flipping the tablet to make the ball moves towards the desired point. Therefore, this result confirmed the reasonable assumptions on the game’s ability to monitor these types of skills. *Sharing*, on the other hand, disclosed an unexpected potential in this area.

Progress in social and emotional skills turned out to be recognizable by the *Cat and Dog* game. The result obtained in this case was the highest - nearly 89%. *Cat and Dog* was the most difficult and it was predicted to reveal progress in more advanced skills. Some children refused to play it at all, while others tapped at the screen whenever a new object appeared. Obviously, these were individuals who did not understand the rules. But whenever a child understood the game it involved him/her deeply. This particular feature of the game affected the type of data obtained. Only the data from some children were suitable for training the classifiers, so the result was obtained for a less representative group.

The highest accuracies in the case of stereotypical behaviours were obtained on the basis of data from *Pinwheel* game −80%. This game was exceptional in the sense of control as it required tilting the tablet. None of the children had ever had opportunity to use a tablet in this way and it turned out to be really difficult. Therefore only some of them, usually the older and better functioning ones, managed to play the game.

Three of the games–i.e. *Sharing*, *Creativity*, *Cat and Dog*–showed some potential for recognizing progress in reacting to stimulation, with recognition accuracies of around 78–80%. Sharing was specially designed to test players’ inclination towards reacting to distractors. A number of objects placed on the screen attracted some players’ attention and led them to break the game rules. In *Cat and Dog*, appropriate reaction to stimuli, either images or sounds was the goal of the game.

In the case of attention control, *Sharing* achieved the highest score of nearly 82% accuracy. Although all the games require attention control, *Sharing* was the game all children enjoyed and thus most of them played it for a long time. *Sharing* food in the same way for five minutes seems rather boring, but attractive graphics and sounds diminished this particular drawback. The children spent the largest amounts of time playing it, often until the game’s time limit.

The proposed method does not seem to be able to recognize therapy progress in the following three areas of development: challenging behaviours, self-reliance and following instructions. Challenging behaviours in fact were never expected to be one of the best candidates for the proposed application, but the other two areas definitely were, especially the ability to follow instructions.

Recognition accuracies, however important, should not be analysed alone. Other parameters, such as recall, which denotes the probability of correct prediction in a particular class and precision denoting the probability that a particular prediction is correct, need to be taken into account. Even if the overall accuracy is high but the recall parameter, for example, is low, the model is useless. Taking this into account and the results presented in Table 3, the following conclusions on the games and their most appropriate applications may be drawn: *Boxes* correlates well with therapy progress in the area of fine motor skills; *Sharing* in the area of fine motor skills, gross motor skills and attention control; *Creativity* in reacting to stimulation; *Pinwheel* in gross motor skills. None of the games seems to be a universal tool for all areas.

The data used for training was preprocessed using normalization, principal component analysis, or both procedures. The preprocessing stage influences the final models. The differences in the results obtained for sets of data preprocessed in different ways were high in some cases. Although it is impossible to clearly indicate the optimal rule for data preparation, it may be noticed that using the features extracted in the process of principal component analysis usually improves the classifiers’ effectiveness. Normalization in some cases did not change the original data much, because most features were designed as normalized, usually by the game time.

### Feature evaluation

**Figure 2:**
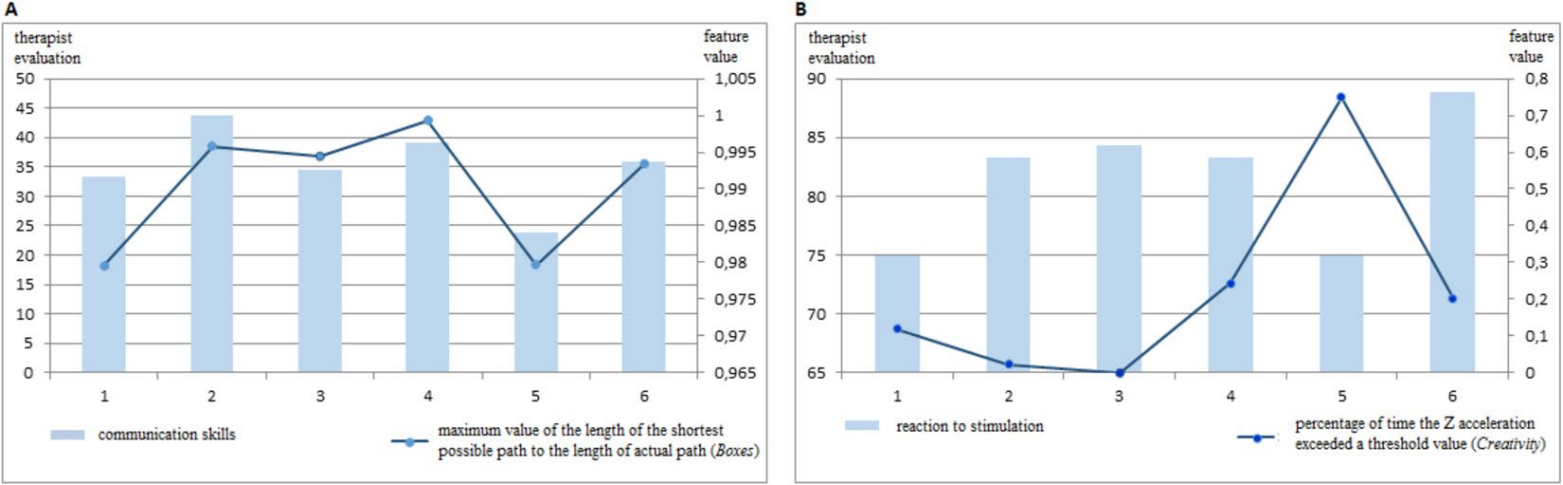
Example features (lines) correlating well with therapists’ evaluations (bars): (**A**) a feature positively correlating with the evaluation of communication skills, (**B**) a feature negatively correlationg with the evaluation of reaction to stimulation.

The parameters extracted from raw data were analysed in order to identify the best progress indicators. Two steps of feature analysis were performed. First of all, two types of correlation coefficients - i.e. Pearson’s linear and Spearman’s rank - between the value changes of these parameters and the value changes of the therapists’ evaluations were calculated. Figure 2 presents two examples of feature values which correspond well with the therapists’ evaluations. Each graph shows the results of six subsequent sessions of a particular child. The dots present a feature value and the bars correspond to the therapists’ evaluations in a given area of development. Figure 2A shows one of the features calculated on the basis of data from the *Boxes* game. There is a positive correlation between the values it yielded and the evaluation in the area of communication skills. Figure 2B reveals a negative correlation between a feature from the *Creativity* game and the evaluation in the area of reacting to stimulation. This example shows two of the parameters with the highest correlations. One of the experiment objectives was to identify a set of such parameters for each area of development. A list of features that correlate the best with the therapists’ evaluations has been presented in Table 4. Depending on the area of development, different numbers of features have been found. In the case of communication skills there are nine of them originating from four out of five games. Therefore, the tool seems to be useful in indicating progress in communicative ability. No parameter could be selected in the area of following instructions, thus confirming the unsatisfying results for therapy progress recognition obtained in this area as illustrated by Table 3.

**Table 4:** A subset of features selected on the basis of correlation index or information gain.

Area of development	Feature	F – flow T- touch I - inertial	Game
communication skills	total game time	F	Boxes
	number of ball moves	F	
	number of paths	F	
	maximum value of the length of the shortest possible path to the length of actual path	T	
	the number of unsuccessful placements in a box	F	
	percentage of time the tablet was tilted forward	I	Pinwheel
	maksimum moving speed	T	Sharing
	minimum speed of path drawing	T	Creativity
	minimum vertical range of colour filling paths	T	
gross motor skills	the number of successful moves divided by the total number of moves	F	Boxes
	maksimum moving speed	T	Sharing
	number of successful hits divided by the total time	F	Pinwheel
fine motor skills	total game time	F	Boxes
self-reliance	vertical range of colour filling paths (mean, standard deviation, median, maximum)	T	Creativity
	maximum length of colour filling paths	T	
	minimum moving speed for food pieces	T	Sharing
	the number of short moves ending with ball placement in a box (both successful and unsuccessful)	F	Boxes
social and emotional skills	minimum and median value of the length of colour filling paths	T	Creativity
	minimum horizontal range of colour filling paths	T	
stereotypical behaviours	maximum and mean reaction time for correct image touch answers	F	Cat and Dog
	mean reaction time for all image touch answers	F	
	percentage of time the X acceleration exceeded a threshold value	I	Creativity
reaction to stimulation	percentage of time the X acceleration exceeded a threshold value	I	Creativity
	percentage of time the Z acceleration exceeded a threshold value	I	
	minimum reaction time for correct image touch answers	F	Cat and Dog
	minimum reaction time for all touch answers	F	
	median value for left tilt oscillations measured as the number of local extremes for a tilt	I	Pinwheel
attention control	mean value and standard deviation for maximum forward tilts	I	Pinwheel
	maximum vertical range of colour filling paths	T	Creativity
challenging behaviours	the intensity of drawing horizontal paths measured by changes of X acceleration sign	I	Creativity
	number of plate → plate moves for distractor objects divided by the total time	F	Sharing

Apart from analysing the relations between the feature values and therapists evaluations, it is also possible to take into account the final classification labels (*progress*/ *no progress*) to estimate the features’ relevance in terms of evaluating therapy progress. Information gain, which is a commonly used criterion in feature selection, was calculated for each of the 181 features. Eventually, for each area of development, some features, either correlating the best with the therapists’ evaluations or revealing the highest information gain, were selected as the best progress indicators for this area. A list of these features are presented in Table 4. The set of selected parameters might be treated as progress predictors and used to visualize the course of therapy in a tool designed for therapists.

### Validation

Finally, the proposed methods were subjected to validation. The recipients of the created tool, - i.e. the therapists - were asked to complete a questionnaire to evaluate the level of understanding of the proposed metrics, their usability and efficacy of presentation. The usability was evaluated with regard to reporting the effects of the therapy, verifying the effectiveness of the therapy and modifying the therapy plan. Moreover, they were asked to indicate other potential applications of the method. 20 therapists filled in the questionnaire. 17 of them declared that they would like to use the application to support the measurement of therapy progress. Some of the metrics turned out to be more user-friendly than others. The metrics evaluated by 75% of the therapists as the most comprehensible are: effectiveness in a game and reacting to distractors as a measure in the area of stereotypical behaviours, reaction to stimulation and challenging behaviours. The least understandable metric was the tilt variability recorded during the *Pinwheel* game. Incomprehension of the assumptions of some metrics and their, sometimes inadequate, assignments to particular areas of development was pointed out as a drawback of the proposed solution. As one of the main advantages the therapists indicated the way results are presented as well as the possibility for automated data collection and processing. Moreover, they highlighted that, from a child’s point of view, the proposed solution is an exceptionally friendly method of monitoring progress.

## Discussion and study implications

The study results indicate that changes in behavioural characteristics derived from mobile games can be identified by machine learning. The study confirms the possibility of measuring the motor patterns of autistic children via mobile devices. This finding is in line with previous studies that used the characteristics in order to identify children with autism^26^. However, the investigated patterns (autism motor signature) might be not consistent over time. In this study we identified a number of features that change along with a child’s progress, which constitutes a novel contribution to further investigations in both autism recognition and progress monitoring. The study reveals that long-term (a minimum 3-month period) changes in skills and deficits correlate with changes in behavioural characteristics measurable via mobile games.

Nevertheless, the previous study used two types of features derived from touch and inertial sensors^26^. In our study we employed three feature types: touch, inertial and derived from game and interaction flow. The results indicate that among the features with the highest information gain (see Table 4 for details), there were 11 touch-screen-based features, 7 inertial characteristics and 13 features corresponding to interaction flow. A benefit of using flow features is that they are less device-dependent and easier to monitor and calculate. However, most of the flow characteristics are game-dependent.

Furthermore, the presented study demonstrates that the behavioural patterns reflect not only fine motor skills development, but also the development in other areas. One of the previous studies revealed that motor patterns were related to communication skills^29^. Our study adds to that observation, illustrating that progress in communication was recognized with accuracies varying from 67% up to 85% depending on the game. In another study, the authors identified the challenging relationship between kinematics and social perception and interaction^24^. In our study, progress in social skills was recognized with accuracies varying from 65% up to 88% depending on the game. Also gross motor skills progress was demonstrated with accuracies up to 79%. The correlation between motor skills and the behavioural characteristics of interaction with mobile device is intuitive, although the prediction of other developmental areas is not surprising, taking into account previous research on the nature of motor disturbance in autism. The complex aetiology of autism points to genes, metabolites and neuroanatomy. As a result, the sensorimotor information processing in autism might be a common factor that influences both motor patterns and other developmental areas, which results in the unveiling of a correlation among them^23,49–51^.

In terms of limitations of the present study, we were unable to exclude gender as a potential confounder - only 9 females took part in the study. The inequality between male and female group is rather typical in autism-related studies due to the autism gender distribution pattern. Furthermore, the participants were only recruited from therapy centres located in Poland (10 centres agreed to take part in the study), which means that the study does not necessarily reflect the characteristics of the whole population of children with autism. Replication of the study in other countries might be beneficial in order to make more general conclusions. Additionally, the sample size is not exhaustive. Although the authors spent more than six months on the data collection phase and gathered data from 40 individuals, not all the data samples were suitable for the experiment. Sometimes a child refused to play a game; sometimes the game time was too short to record enough data. Moreover, not all the children took part in a session every month. Therefore, the series of parameter values were of different lengths or the time between two subsequent sessions was not always the same. All these issues in turn reduced the size of the training sets, which was small if compared to the number of features, especially in the case of recognizing progress between the first and the last session.

The method of assigning labels might be another validity threat. The strategy of calculating the evaluation in a given area of development, was based on averaging the evaluations of particular elements. No weights were assigned to these elements. Moreover, the set of evaluated elements was agreed with therapists from several therapy centres and must have been influenced by the approach used in their work, as different centres apply various evaluation approaches. Two rounds of interviews with the therapists were arranged to finally establish the contents of the evaluation questionnaire. Although the selection of the evaluated elements was made in a deliberate, iterative process, one may find the selected subset to be biased.

Another validity threat that was inevitable in this study design, was a possible history effect. One might expect the children to become more and more proficient in using the given application, which in turn would influence the data gathered from the games. However, the authors claim that contact limited to a few minutes once a month should minimize such threat. Earlier studies performed by the authors, in which no history effect during subsequent sessions was found, confirm this assertion^35^.

Finally, there are numerous issues, which may affect a child’s behaviour. This is inevitable in a real environment, especially with individuals with autism, whose behaviour may change unexpectedly according to circumstances. This is especially visible among those who display challenging behaviours. Any absence, caused by an illness for example, might also disturb the therapy rhythm. Although it was not possible to eliminate such situations, they were all noted and a decision was made whether or not a data sample needed to be discarded from the training set.

The results presented in Table 3 refer to the recognition of progress between the first and the last session a child took part in - i.e. after a few month period. Although another analysis was performed on recognizing progress between two subsequent sessions - i.e. after one month -the results obtained in that case were not satisfactory and therefore not mentioned in this paper.

To sum up, the authors believe more data and more analysis would improve the final models able to predict therapy progress. The practical implications offered by the presented study for therapy include the following:

- specialized games might be used in therapy to unobtrusively measure children’s progress over long-term periods;
- according to the therapists who validated the solution, the metrics might be used for better progress estimation along with existing manual annotations;
- the behavioural metrics obtained via mobile devices might be used for reporting the progress of a child in a more measurable manner.

The obtained results confirm the stated hypothesis, that it is possible to recognize therapy progress among children with autism in some areas of development. We have shown here that mobile technology offers an attractive option for monitoring children’s developmental progress. Tablet games might be an ecological and engaging method for investigating progress in motor skills as well as other developmental areas. Accessible and natural, the method might be implemented as a tool designed for therapists, which could add value to their everyday work by tracking the course of the therapy and recommending further actions. Any improvement made in the process of therapy may influence its results and thus help children with autism to achieve independence in adulthood.
